# What Results Should Be Returned from Opportunistic Screening in Translational Research?

**DOI:** 10.3390/jpm10010013

**Published:** 2020-03-01

**Authors:** Colin M. E. Halverson, Sarah H. Jones, Laurie Novak, Christopher Simpson, Digna R. Velez Edwards, Sifang Kathy Zhao, Ellen W. Clayton

**Affiliations:** 1 Center for Bioethics, Indiana University School of Medicine, Indianapolis, IN 46202, USA; 2 Regenstrief Institute, Indianapolis, IN 46202, USA; 3 Vanderbilt Epidemiology Center, Vanderbilt University Medical Center, Nashville, TN 37235, USA; sarah.h.jones@vumc.org (S.H.J.); digna.r.velez.edwards@vumc.org (D.R.V.E.); sifang.zhao@vanderbilt.edu (S.K.Z.); 4 Department of Biomedical Informatics, Vanderbilt University Medical Center, Nashville, TN 37235, USA; laurie.l.novak@vumc.org (L.N.); chris.simpson@vumc.org (C.S.); 5 Institute for Medicine and Public Health, Vanderbilt University Medical Center, Nashville, TN 37235, USA; 6 Vanderbilt Genetics Institute, Vanderbilt University Medical Center, Nashville, TN 37235, USA; 7 Division of Quantitative Sciences, Vanderbilt University Medical Center, Nashville, TN 37235, USA; 8 Department of Obstetrics and Gynecology, Vanderbilt University Medical Center, Nashville, TN 37235, USA; 9 Center for Biomedical Ethics and Society, Vanderbilt University Medical Center, Nashville, TN 37235, USA; 10 Department of Pediatrics, Vanderbilt University Medical Center, Nashville, TN 37235, USA; 11 School of Law, Vanderbilt University, Nashville, TN 37235, USA

**Keywords:** clinical utility, personal utility, genetic testing, genomic testing, return of results

## Abstract

Increasingly, patients without clinical indications are undergoing genomic tests. The purpose of this study was to assess their appreciation and comprehension of their test results and their clinicians’ reactions. We conducted 675 surveys with participants from the Vanderbilt Electronic Medical Records and Genomics (eMERGE) cohort. We interviewed 36 participants: 19 had received positive results, and 17 were self-identified racial minorities. Eleven clinicians who had patients who had participated in eMERGE were interviewed. A further 21 of these clinicians completed surveys. Participants spontaneously admitted to understanding little or none of the information returned to them from the eMERGE study. However, they simultaneously said that they generally found testing to be “helpful,” even when it did not inform their health care. Primary care physicians expressed discomfort in being asked to interpret the results for their patients and described it as an undue burden. Providing genetic testing to otherwise healthy patients raises a number of ethical issues that warrant serious consideration. Although our participants were enthusiastic about enrolling and receiving their results, they express a limited understanding of what the results mean for their health care. This fact, coupled the clinicians’ concern, urges greater caution when educating and enrolling participants in clinically non-indicated testing.

## 1. Introduction

Many diagnostic interventions—from taking a history to performing tests—are devoted to answering specific questions driven by the patient’s individual situation: What is the cause of the patient’s current problem? Is the patient at risk for a particular disease that could be ameliorated by action in the present? Would one therapeutic or preventive intervention be more effective or less toxic than another? When the answer is affirmative, the results of these questions may be called “actionable,” in that obtaining it will make a difference in current medical care [1]. Health care professionals are taught not to order a test if it is not expected to aid in diagnosis or alter management [2]. Guidelines for screening invoke a similar calculus: does the impact of early detection on the person’s long-term clinical outcome justify the individual and social costs of screening? [3].

Yet genomic tests can contain more information than is needed to answer the clinical question that led to testing in the first place. There has been much debate about how much effort should be made to analyze and return these additional data in both the clinical and research settings [1,4]. Many arguments have been proposed for returning these additional individual results from genetic tests: duty to rescue [5], respect for persons [6], or simply because participants want these results [7]. Moreover, in research settings, some scholars endorse returning results to encourage research participation [8].

Although most commentators urge returning only actionable results, some scholars argue against using actionability as a criterion. They contend that patients are entitled to information even if it does not inform their own medical care [9]. Reasons include that participants may want to share the information with family members or they may simply want to know it, without any particular intention to utilize it. These reasons have often been characterized as “personal utility.” While some scholars argue that the designation requires that it be reasonably possible to be put such information to use [10], other commentators have equated personal utility with peace of mind, with the desire to contribute to the progress of science, and with general curiosity [11]. Some urge that benefits should be viewed solely from the individual’s subjective perspective [7]. Numerous studies suggest that people value genetic test results in exactly these ways [12]. All of these arguments, however, assume that recipients derive some meaning from the results.

The increasing availability of multiplex tests that detect genetic alterations across the genome provokes a debate about the appropriate weight to be placed on personal utility when deciding whether to offer broad-based tests and what results to return. In the clinical setting, pressure is growing to use genome-based approaches instead of more targeted testing—even when the patient has a specific medical problem—reflecting a perception that multiplex testing is more cost-effective and will be a resource in the future [13]. Some have argued that in genetic medicine, it is appropriate and even mandatory to look beyond the results needed to answer the clinical question that initially prompted testing [14].

In the research setting, many participants who are otherwise healthy (or at least were not recruited for their known risk for a particular disorder) undergo broad-based testing, ranging from genetic sequencing in MedSeq [15] and BabySeq [16] to tests for variants in up to 109 genes in the Electronic Medical Records and Genomics (eMERGE) consortium, discussed herein. The likelihood that a test result will inform clinical care often depends on whether the participant has a specific indication for inclusion—as was often the case of Clinical Sequencing Evidence-Generating Research (CSER) [17]—or whether testing was opportunistic in that participants had no prior indication for testing—as in the case of MedSeq. Here as well, the question of which results ought to be sought by investigators and returned to participants is highly contested. 

Significant burdens are placed on clinicians who have limited time and resources but nonetheless are asked to return and interpret participants’ results that the participants’ clinicians had not ordered. These costs must also be considered when setting criteria for which results to disclose and how to do so. Non-specialist clinicians sometimes feel unprepared to address these issues [18] and worry about the effect of returning these results on their time and the workflow of their clinics [19].

In order to address these questions, we report here the eMERGE experience at Vanderbilt University Medical Center (VUMC). The eMERGE Network is returning research results to 20,000 participants—most of whom were recruited opportunistically. The study is thus a prototype of the present and near future, where people will get multiplex tests on their own, as a product of research participation, or even as part of clinical care, and where testing may be ordered by clinicians who seek only some of the results to be returned. 

For our analysis, we explore the question of how research participants perceive the value of genetic testing. We looked not only at their motivations to pursue testing and their evaluation of the results, but also their report of the effects that the results have had on their behavior and understanding. We thus asked not only how participants in Vanderbilt’s eMERGE study value their participation, but also what constituted that value, concretely. We evaluate these findings in light of prominent, competing perspectives on personal value. We also asked clinicians who received participants’ results from eMERGE what getting these results meant both for the participant and for their practice. 

## 2. Materials and Methods

Vanderbilt’s cohort in the eMERGE study includes 2453 participants who have been tested for variants in 109 “actionable” genes, including the American College of Medical Genetics and Genomics’ list of genes recommended for reporting as secondary findings in clinical care [20]. All results, both positive and negative, were returned to the participants’ primary care physicians and placed in their electronic health record. Participants found to have pathogenic or likely pathogenic variants were notified by phone and received additional specialist counseling at that time or at a later clinic visit. All participants received a letter written at an eighth-grade level that included their results. A copy of the laboratory report was included with the letter. 

Almost two thousand participants were recruited to eMERGE at VUMC from the PREDICT (Pharmacogenomic Resource for Enhanced Decisions in Care and Treatment) cohort (*n* = 14,500) [21]. The eMERGE study also utilized My Research at Vanderbilt, the Personalized Medicine Biorepository (IRB# 150435), and direct recruitment from outpatient internal medicine clinics using Subject Locator [22]. Several participants were identified using phenotype risk scores [23].

Data for this paper come from two sets of interviews and two sets of surveys conducted with eMERGE participants at VUMC and the clinicians who received results (IRB## 150209, 170501, 180422, and P00028295, Boston Children’s P00028295). All interviewees were offered a $25 gift certificate in appreciation for their time, while survey participants received a $10 prepaid Comdata card for completing the survey. The interview guides and the domains covered in the surveys are available in the online supplement.

### 2.1. Participant Surveys and Interviews

All participants who had a valid email address—regardless of their test results—were invited to complete a survey in REDCap (a web-based application used to manage online surveys and databases). The participant survey was developed by the eMERGE III ELSI Working Group (WG). It contained up to 121 questions, depending on selections that triggered branching logic, examining individuals’ experience as participants in the eMERGE study, their reactions to receiving results from the genetic test, their plans to use and share the information they had gained, as well as their views about privacy. 

An in-depth, semi-structured interview guide for participants [available in the online supplement] was developed by our research team after reviewing existing literature and in consultation with other methodologists and skilled qualitative researchers at VUMC. The participant interview guide addressed all the major topics of the eMERGE survey and considered questions of privacy in greater depth and obtained additional demographic information. Two study staff recruited interviewees from participants who were at least 18 years of age and spoke English. We purposively oversampled African Americans, as this population has historically been underrepresented in similar studies [24], hypothesizing that they might have distinctive responses.

### 2.2. Clinician Surveys and Interviews

The second set of sources for our data were the responses of Vanderbilt clinicians. Invitations to a REDCap survey created by the WG were sent by email to Vanderbilt clinicians whose patients had received a positive result from eMERGE. We also sought to conduct telephone interviews with the same set of clinicians. One of the authors (EWC) reached out to individual providers by email or phone up to three times. The clinician interview guide was developed in the same manner as that described for the participant cohort, informed by earlier interviews, and is available in the online supplement [19].

### 2.3. Data Analysis

Survey and interview data for eMERGE participants were analyzed and demographic characteristics were summarized in Table 1. Survey responses were compared between participants with and without positive results (Table 2). Logistic regression was used to calculate odds ratios (ORs) and 95% confidence intervals (CIs) to determine the association between receiving positive results and participant responses (Table 3). Ordinal logistic regression was used for ordinal values. A priori confounders of age (continuous), sex (male vs. female), and race (White vs. African Americans) were included in all adjusted models. We also used logistic regression to investigate whether age, sex, race, and result type were associated with having no emails or not responding to the survey. All statistical analyses were performed at a 2-sided significance level of 0.05 using STATA 14.2 (StataCorp, Texas, United States). 

All interviews were conducted by one of four experienced interviewers trained in qualitative methods (CMEH, LN, CS, EWC). Interviews were audio-recorded and transcribed verbatim. Identifying information was removed and replaced with a randomly assigned three-digit pseudonym, and the transcripts were uploaded into Dedoose, a web-based mixed-methods analysis tool, where they were coded by multiple researchers (CMEH, LN, CS) using a coding tree that was developed iteratively based on discussions of emerging themes. 

## 3. Results

### 3.1. Response Rate and Demographics

**Figure 1:**
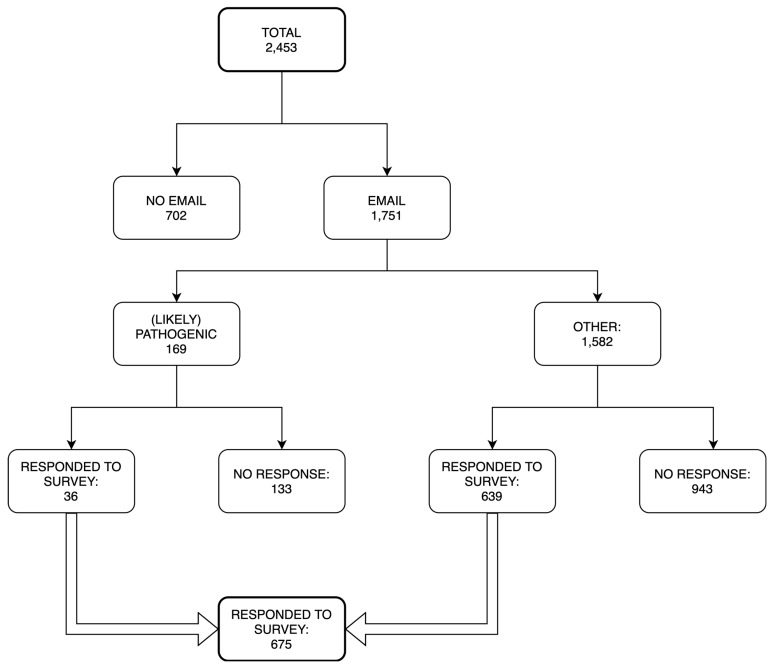
Survey recruitment process.

Among 2453 VUMC participants enrolled in the eMERGE study, email addresses were available for 1751 (71.4%). Participants who were older (OR 0.96, 95% CI 0.95, 0.97) and African American (OR 0.46, 95% CI 0.32, 0.66) were less likely to have an email address. Of those eligible participants, 675 completed the REDCap survey—36 (5.3%) of whom had received a pathogenic or likely pathogenic variant. (See Figure 1.) Among those with email addresses (*n* = 1751), males (OR 0.76, 95% CI 0.62, 0.93) and participants with positive results (OR 0.39, 95% CI 0.27, 0.58) were less likely to respond. (By positive results, we refer to pathogenic or likely pathogenic results and do not include pharmacogenetic results.) Additional breakdowns of demographic characteristics by responding status and result type are shown in Supplemental Tables S1–S3.

**Figure 2:**
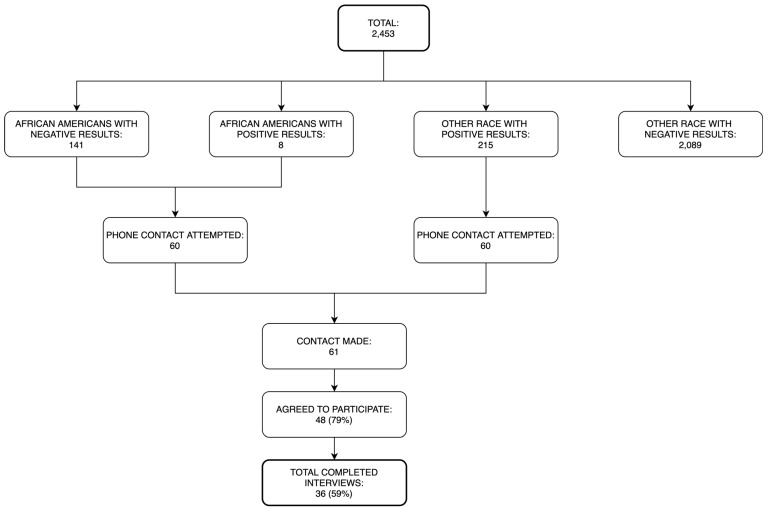
Interview recruitment process.

In order to conduct interviews with eMERGE participants, we randomly selected and attempted phone contact with 60 African Americans and 60 non-African Americans with positive results. We reached 61 eMERGE participants by phone and 36 (59%) completed the interview. (See Figure 2.) Of these individuals, seventeen were African Americans and nineteen were either white or Hispanic with pathogenic or likely pathogenic results in one or more of the 109 targeted genes (Table 1). Notably, all of the African American interviewees had received only results about pharmacogenomic variants. 

Twenty-one (37%) health care providers completed the online survey out of 57 initially invited because one or more of their patients had received a positive result. Thirty-one clinicians were invited to complete an interview, but only 11 (35%) agreed to participate. All clinician interviewees were employed at VUMC and had seen at least one eMERGE participant whose test results were positive. Nine (82%) of these interviewees were primary care physicians.

### 3.2. Survey Participants’ Experience 

Overall, 68% of participants viewed the privacy and security aspects of genetic information as the same as other health information, and 74% said they were willing to share the results with one or more family members, and most planned to or already had made lifestyle changes (Table 2). According to the decision regret scale [25] and FACToR (an instrument for measuring the psychosocial impact of returning genetic test results) [26], overall, respondents reported low levels of decision regret, negative emotions, uncertainty, or concerns about privacy. More specifically, 95% believed that it was good that they had participated, 93% had no regrets about participation, 96% said they would do it again, 89% called it a “wise decision,” and 76% said the information they received from genetic testing was at least a little helpful in planning for the future.

When further stratified by result type, we found that participants’ experience with enrollment and receiving results from eMERGE differed based on whether the result was positive or not (Table 3). Adjusting for age, sex, and race, those who had received positive results stated that they were more willing to talk with their physicians (OR 9.2, 95% CI 4.4, 19.1), to have more negative emotion (OR 4.2, 95% CI 2.2, 8.1) and less positive emotion (OR 0.5, 95% CI 0.3, 1.0) about their results, and to view genetic information differently from other medical information (OR 2.4, 95% CI 1.2, 4.9). They, however, did not express any difference in their confidence about privacy and confidentiality or in their level of regret about participating.

### 3.3. Interviewees Found Results “Helpful” 

Our interviewees were similarly enthusiastic about participation, with only five (14%) saying that they found genetic testing to have been “not helpful” for them. The majority was glad they participated, and none expressed regrets. One woman who had not received any positive results explained why she found participation helpful: “It didn’t really change what I had probably planned to do; it just helped ease my mind” (125). Few stated that they had done anything with the results, even though 53% had received positive results. 

### 3.4. Interviewees Often Did Not Understand Results

Participants received a letter describing the meaning of their results in addition to their laboratory report. Six interviewees (17%) stated they understood and greatly appreciated the return of their results. Nonetheless, many respondents remained confused by their results. Fifteen interviewees (42%)—of whom one-third had received positive results—spontaneously admitted they understood little of the significance of what had been returned to them. For instance, one woman had her laboratory report in front of her throughout the interview. She said she could not even discern which portion of the report was meant to display her results. 

One man received only pharmacogenetic results from his genetic test. He had not spoken with his primary care physician about the test but told the interviewer that he planned to bring the subject up at his next regularly scheduled appointment. When asked whether he found it helpful to get back his genetic test report, he said yes. 

Interviewer: And can you tell me in what way?

Respondent: Just to kind of see some of the numbers and what they did, even though I didn’t fully understand it. (…) It was just a good chance to get my numbers and the DNA.

Interviewer: And when you say ‘numbers,’ what are you specifically referring to?

Respondent: I mean, I didn’t really understand it. I [do] remember seeing the numbers on the report.

Even though he felt testing had been “helpful,” he could not express how it might help him. 

A different type of confusion was demonstrated in an interview with a woman who had received a pathogenic variant linked to Long QT Syndrome. She had, in fact, been diagnosed with the disorder several years before enrolling in eMERGE, but she said she had “no idea” what results she had received from the study. When asked what she meant when she said the results were “not interpretable,” she responded: “Not by us mere mortals.” 

### 3.5. Clinicians Often Felt Uncomfortable and Overburdened 

Clinicians had different perspectives about the value of the eMERGE process. Although all clinician interviewees had seen participants with positive results, only four (36%) believed that testing had been beneficial for the participants. Many worried about their own ability to interpret the results, the amount of time added to their workflow, and issues of insurability and discrimination the participants might face. “I had to have conversations with the eMERGE physicians; I had to look stuff up; I had to personally reach out to the patient and have a conversation with the patient. It created a lot of additional work,” one primary care physician enumerated. Three other clinicians we contacted said they refused to return results to their participants at all, believing it to be an undue burden on their practice and requesting research staff take on this responsibility. (These participants were contacted by eMERGE staff regarding their results.)

Clinicians’ responses to receiving genetic test results from this study revealed three distinct perspectives. The first consisted of clinicians who simply referred the participant to a specialist to interpret the report. These clinicians were the most positive about their experience with eMERGE, viewing the results as valuable to the participants and/or the process as not overly burdensome. The second consisted of clinicians who received a molecular diagnosis for a participant who had already been clinically diagnosed with that disorder. These clinicians expressed frustration at having to interpret the results for participants who already knew the diagnosis; they did not find testing helpful. The third personally counseled the participant. Their responses about the utility of the testing were mixed, although most complained about the amount of time it added to their already busy schedules. Of the four clinicians who said they thought genetic testing had been helpful, three counseled the participant themselves. One referred the participant to a specialist. 

The clinicians who responded to the survey were also ambivalent. While most thought the results were important to the participant’s health, almost half worried that participants would be harmed by unnecessary testing. The majority (81%) said they had been only a little or not at all confident that their training had prepared them to explain the genetic test result. Nearly three quarters (72%) said they were only a little or not at all confident in their ability to explain the specific eMERGE result they were asked to interpret, and 78% said they were only a little or not at all confident in their ability to answer the participant’s questions about the result. Nearly half (45%) said that responding to the eMERGE result was burdensome. Even so, three quarters said they would like to receive unsolicited genetic results that showed no risk or suggested no effective medical interventions.

## 4. Discussion

In the VUMC eMERGE protocol, results were returned to all participants and their primary care providers, responding to reports that people want this information [27,28] and arguments that such return is ethically warranted [6]. We assessed the impact of returning these results in a real-world setting. While participants were generally quite enthusiastic about receiving results, a large majority did not plan to discuss them with their providers even when they had received positive results. Most participants indicated that they planned to make lifestyle changes, although previous studies demonstrate that this rarely happens [29]. In our follow-up interviews, few of the respondents understood their results, even when they had been individually counseled.

Participants valued their test results and believed that they were “helpful.” They were happy to have participated in eMERGE and neither regretted having done so nor experienced adverse sequelae, which is consistent with other studies [30,31]. However, characterizing the results as helpful does not necessarily mean that they were clinically relevant, as most participants received results that were not considered actionable. Additionally, many participants explicitly stated that they did not understand their results, suggesting again that the “helpfulness” of the results was not intrinsically connected to the reason a clinician might order a genetic test. Patients’ and research participants’ lack of understanding—or explicit misunderstanding—is common enough, at times raising questions about inappropriate reassurance, particularly in opportunistic screening [32]. Our participants’ enthusiasm was rooted in excitement about things other than the results’ clinical utility. 

Participants’ optimistic evaluations of the return of results could be positive signs if viewed from the perspective of personal utility [10,12]. They are concordant with studies that have suggested that individuals often desire genetic information even in the absence of clinical relevance [27,28] and do not experience anxiety from return of such results [33]. Yet it is important to specify concretely what personal utility means, especially with regard to its relationship to clinical utility. While our participants were generally happy about their participation and even called their results “helpful,” many readily said they understood little to nothing of what their results meant for their health or health care. Even though our participants appreciated the opportunity to engage in this type of research, we question whether that value sufficiently counterbalances the limitations of such results and the burdens they place on clinicians. We agree with Bunnik and colleagues’ argument that “there can be no personal utility in uninterpretable information” [10]. It is not clear what many of our participants found valuable beyond the return of results itself. It is clear, however, that what many derived from those results was not clinically relevant information. 

Some have argued that returning individual research results is an ethical obligation, demonstrating a respect for persons [6]. Advocates of this perspective contend that declining to return such results relegates participants to the status of means-to-an-end, denying them the potential benefits of the data that their participation helped to generate [3,5]. More recently, some scholars have argued that returning results is important to motivate enrollment [8].

We do not disagree that receiving information about themselves may stimulate many individuals to participate in research that they would have otherwise forgone. Neither do we disagree that returning clinically relevant data to participants can be valued by recipients. However, a critical assumption in this argumentation is that returning results would benefit the participant in some way related to the research project itself. Data that are uninterpretable by the participant (because they are uncertain or not clinically significant, or because they are not presented in a legible way) cannot benefit participants in that way. Expressing an interest in genetic information without the intent or ability to use it to make decisions related to their health and health care is not sufficient to support even a weak obligation to return results. 

Moreover, returning results—especially results that are not clinically relevant—is burdensome for clinicians, especially those who are not well-versed in genetics. Even when the time added is only minimal, clinicians in these situations often already feel overly put-upon by daily tasks [34]. Many clinicians working in primary care feel uncomfortable returning these results [18,19] and do not feel that it is their responsibility [35], especially when the results are not considered medically actionable [19].

Given what participants thought about their results, it seems appropriate to ask whether returning them is worth the effort. After all, returning individual genetic research results to participants and their clinicians is labor intensive—at VUMC, it required thousands of person-hours—and often required surmounting a variety of hurdles. Moreover, as we showed previously [19], clinicians who care for these participants varied widely in their views about whether these results were helpful for participants. Most noted that receiving these results significantly affected their workflow, and a few refused to return results, saying that research personnel should perform this function.

Our analysis has some limitations: the eMERGE research protocol made testing free for participants. Had they been asked to pay for their testing, would they still have evaluated it so positively? Other studies have suggested that patients do consider financial burdens in deciding to pursue personal utility [36,37]. They are often disappointed that the tests did not reveal more [38]. Another limitation is that our data are taken from only one site. Additionally, ours was a convenience sample largely derived from individuals who had already agreed to participate in a genetic study. Our study does not capture those with no emails, although many had access to the internet, bespeaking a specific sociocultural milieu. They were also older than the general population on average.

## 5. Conclusions

Genomic tests can be powerful predictive and diagnostic tools. However, our study underscores serious issues that must be addressed for such testing to be implemented ethically, including challenges of conveying and acting upon this information, which in most cases should not alter medical care. Both ethically and practically, the primary question is whether the purpose of translational research is to inform the use of these tests in clinical care or to provide information to consumers [39,40] whose wishes may not be well informed and may not align with notions of clinical utility that govern practice. Given the balance of benefit and burden, we suggest that trials of opportunistic screening return at most only pathogenic and likely pathogenic results.

## Figures and Tables

**Table 1:** Demographic characteristics for interview and survey among Electronic Medical Records and Genomics (eMERGE) participants.

	Total (*N* = 2453)		Interview (*N* = 36)		Survey (*N* = 675)	
	*n*	% ^1^	*n*	% ^1^	*n*	% ^1^
Sex						
Male	1308	53.3	16	44.4	333	49.4
Female	1144	46.7	20	55.6	341	50.6
Missing	1		0		1	
Race						
White	2162	92.4	19	52.8	607	93.4
African American	149	6.4	17	47.2	33	5.1
Hispanic	30	1.28	0	0	10	1.5
Missing	112		0		25	
Age at participation ^2^						
≤65 years	1041	42.4	12	33.3	324	48.1
65–74 years	780	31.8	14	38.9	225	33.4
≥75 years	631	25.7	10	27.8	125	18.6
Missing	1		0		1	

^1^ Percentages exclude missing data and may not add up to 100% due to rounding. ^2^ Age is calculated using date of birth and date of interview or survey participation; if date of participation is unavailable, the median date for survey participation was used (10/20/2018).

**Table 2:** Distribution of survey responses by result type (*n* = 675).

		Total *N* = 675	Negative Result *N* = 639 (95%)		Positive Result ^1^ *N* = 36 (5%)	
	*n*	% or Mean (sd)	*n*	% or Mean (sd)	*n*	% or Mean (sd)
View of privacy/security for genetic vs. other medical info						
Different	214	31.7	196	30.7	18	50.0
Not different	461	68.3	443	69.3	18	50.0
Confidence in privacy and confidentiality ^2^						
Score	675	2.4 (0.6)	639	2.4 (0.6)	36	2.3 (0.6)
Willing to share with any family						
Yes ^5^	499	73.9	467	73.1	32	88.9
No	176	26.1	172	26.9	4	11.1
Willing to discuss with physician						
Yes	104	15.7	84	13.3	20	58.8
No	560	84.3	546	86.7	14	41.2
Missing	11		9		2	
Decision regret scale ^3^						
Score	673	9.4 (12.7)	637	9.5 (12.7)	36	6.4 (11.6)
Missing	2		2		0	
Emotional response to RoR (subscale range) ^4^						
Negative emotion (0–12)	670	3.7 (1.5)	634	3.6 (1.4)	36	5.0 (2.7)
Positive emotion (0–16)	670	8.4 (2.6)	634	8.4 (2.6)	36	7.5 (2.6)
Uncertainty (0–12)	667	5.3 (2.6)	631	5.3 (2.6)	36	6.1 (2.5)
Privacy concern (0–8)	666	4.4 (2.5)	630	4.4 (2.5)	36	3.9 (1.7)
Lifestyle changes in response to RoR						
Yes ^5^	383	57.5	358	56.7	25	71.4
No	283	42.5	273	43.3	10	28.6
Missing	9		8		1	

Abbreviations: sd: standard deviation; RoR: return of results. ^1^ Due to small numbers, pharmacogenomic and positive results are grouped together. ^2^ Responses are summed and averaged on a scale of 1–3; a higher score indicates more confidence. ^3^ Responses are summed and averaged on a scale of 0–100; a higher score indicates more distressed/regret. ^4^ Responses are summed for each subscale, missing responses are assumed to take on the average score for that subscale. ^5^ Plan to or already made any lifestyle changes (eat a healthier diet, exercise more, get a full night’s sleep, start taking vitamins/other supplements, consume less/no alcohol, reduce stress, and stop smoking).

**Table 3:** Crude and adjusted estimates for effect of positive result on survey responses (*n* = 675).

	Crude OR	95% CI	Adjusted OR ^1^	95% CI
Different view of genetic vs. other medical info	2.26	1.15, 4.44	2.44	1.22, 4.87
More confidence in privacy and confidentiality ^2^	0.77	0.43, 1.38	0.74	0.40, 1.35
Willing to share with any family member	2.95	1.03, 8.45	2.75	0.95, 7.96
Willing to discuss with physician	9.29	4.52, 19.1	9.19	4.43, 19.1
More regrets in decision ^2^	0.52	0.25, 1.06	0.59	0.29, 1.22
Emotional response to RoR ^2^				
More negative emotion	4.14	2.18, 7.86	4.16	2.15, 8.07
More positive emotion	0.54	0.30, 0.97	0.53	0.29, 0.98
More uncertainty	1.81	1.04, 3.16	1.69	0.95, 2.98
More privacy concern	0.82	0.42, 1.63	0.83	0.40, 1.71
Willing to make one or more lifestyle changes ^3^	1.91	0.90, 4.04	1.91	0.90, 4.10

Abbreviations: OR: odds ratio; CI: confidence interval; RoR: return of results. ^1^ Adjusting for a priori confounders sex, age (continuous), and race (White vs. African American). ^2^ Responses are summed (and averaged for confidence and regrets in decision); scores are ordinal in nature and thus modelled using ordinal logistic regression. ^3^ Plan to or already made any lifestyle changes (eat a healthier diet, exercise more, get a full night’s sleep, start taking vitamins/other supplements, consume less/no alcohol, reduce stress, and stop smoking).

## Supplementary Materials

The following are available online at https://www.mdpi.com/2075-4426/10/1/13/s1, Table S1: Participant demographics by survey responding status; Table S2: Participant demographics by survey responding status and result; Table S3: Participant demographics by survey responding status and result, excluding non-responders with no email address available (*n* = 702)
