# Supplementary material for: What Results Should Be Returned from Opportunistic Screening in Translational Research?
Source: J Pers Med. 2020 Mar 1;10(1):13. doi: 10.3390/jpm10010013 (PMC7151595; doi:10.3390/jpm10010013)
Supplement: Supplementary file 1 [file jpm-10-00013-s001.zip › supplementary files/Supplemental TABLE 3 final.docx]

## **Supplemental Table 3.** Participant demographics by survey responding status and result, excluding non-responders with no email address available (n=702)

|  | | Responders Positive (N=36) | | | | | | Non-responders Positive  (N=133) | | | | | Responders Negative (N=639) | | | Non-responders Negative  (N=943) | | | |
| --- | --- | --- | --- | --- | --- | --- | --- | --- | --- | --- | --- | --- | --- | --- | --- | --- | --- | --- | --- |
|  | | | | n | | %^1^ | | | n | | %^1^ | n | | | %^1^ | | n | | %^1^ |
| Sex |  | | | |  | |  | | |  | |  | |  | | |  |  | |
| Male | 10 | | | | 27.8 | | 68 | | | 51.1 | | 323 | | 50.6 | | | 533 | 56.5 | |
| Female | 26 | | | | 72.2 | | 65 | | | 48.9 | | 315 | | 49.4 | | | 410 | 43.5 | |
| Missing | 0 | | | |  | | 0 | | |  | | 1 | |  | | | 0 |  | |
| Race |  | | | |  | |  | | |  | |  | |  | | |  |  | |
| Non-Hispanic White | | | 35 | | 100.0 | | 123 | | | 94.6 | | 572 | | 93.0 | | | 849 | 93.1 | |
| African American | 0 | | | | 0 | | 6 | | | 4.6 | | 33 | | 5.4 | | | 51 | 5.6 | |
| Hispanic | 0 | | | | 0 | | 1 | | | 0.8 | | 10 | | 1.6 | | | 12 | 1.3 | |
| Missing | 1 | | | |  | | 3 | | |  | | 24 | |  | | | 31 |  | |
| Age at participation^2^ |  | | | |  | |  | | |  | |  | |  | | |  |  | |
| ≤65 years | 18 | | | | 50.0 | | 61 | | | 45.9 | | 306 | | 48.0 | | | 439 | 46.6 | |
| 65-74 years | 9 | | | | 25.0 | | 46 | | | 34.6 | | 216 | | 33.9 | | | 297 | 31.5 | |
| ≥75 years | 9 | | | | 25.0 | | 26 | | | 19.6 | | 116 | | 18.2 | | | 207 | 22.0 | |
| Missing | 0 | | | |  | | 0 | | |  | | 1 | |  | | | 0 |  | |

^1^ Percentages exclude missing data and may not add up to 100% due to rounding
^2^ Age is calculated using date of birth and date of interview or survey participation; if date of participation is unavailable, the median date for survey participation was used (10/20/2018)
