# Supplementary material for: What Results Should Be Returned from Opportunistic Screening in Translational Research?
Source: J Pers Med. 2020 Mar 1;10(1):13. doi: 10.3390/jpm10010013 (PMC7151595; doi:10.3390/jpm10010013)
Supplement: Supplementary file 1 [file jpm-10-00013-s001.zip › supplementary files/Supplemental TABLE 1 final.docx]

## **Supplemental Table 1.** Participant demographics by survey responding status

|  | | Total  (N=2453) | | | | | Non-responders (N=1,778) | | | | | Responders (N=675) | | |
| --- | --- | --- | --- | --- | --- | --- | --- | --- | --- | --- | --- | --- | --- | --- |
|  | n | | | %^1^ | | n | | | %^1^ | | n | | | %^1^ |
| Sex |  | |  | |  | | |  | |  | | |  | |
| Male | 1,308 | | 53.3 | | 975 | | | 54.8 | | 333 | | | 49.4 | |
| Female | 1,144 | | 46.7 | | 803 | | | 45.2 | | 341 | | | 50.6 | |
| Missing | 1 | |  | | 0 | | |  | | 1 | | |  | |
| Race |  | |  | |  | | |  | |  | | |  | |
| Non-Hispanic White | 2,162 | | 92.4 | | 1,555 | | | 92.0 | | 607 | | | 93.4 | |
| African American | 149 | | 6.4 | | 116 | | | 6.9 | | 33 | | | 5.1 | |
| Hispanic | 30 | | 1.3 | | 20 | | | 1.2 | | 10 | | | 1.5 | |
| Missing | 112 | |  | | 87 | | |  | | 25 | | |  | |
| Age at participation^2^ |  | |  | |  | | |  | |  | | |  | |
| ≤65 years | 1,041 | | 42.4 | | 717 | | | 40.3 | | 324 | | | 48.1 | |
| 65-74 years | 780 | | 31.8 | | 555 | | | 31.2 | | 225 | | | 33.4 | |
| ≥75 years | 631 | | 25.7 | | 506 | | | 28.5 | | 125 | | | 18.6 | |
| Missing | 1 | |  | | 0 | | |  | | 1 | | |  | |

^1^ Percentages exclude missing data and may not add up to 100% due to rounding
^2^ Age is calculated using date of birth and date of interview or survey participation; if date of participation is unavailable, the median date for survey participation was used (10/20/2018)
