# Supplementary material for: What Results Should Be Returned from Opportunistic Screening in Translational Research?
Source: J Pers Med. 2020 Mar 1;10(1):13. doi: 10.3390/jpm10010013 (PMC7151595; doi:10.3390/jpm10010013)
Supplement: Supplementary file 1 [file jpm-10-00013-s001.zip › supplementary files/Supplemental TABLE 1 final.pdf]

Supplemental Table 1. Participant demographics by survey responding status

	Total (N=2453)		Non-responders (N=1,778)		Responders (N=675)	
	n	%[1]	n	%[1]	n	%[1]
Sex						
Male	1,308	53.3	975	54.8	333	49.4
Female	1,144	46.7	803	45.2	341	50.6
Missing	1		0		1	
Race						
Non-Hispanic White	2,162	92.4	1,555	92.0	607	93.4
African American	149	6.4	116	6.9	33	5.1
Hispanic	30	1.3	20	1.2	10	1.5
Missing	112		87		25	
Age at participation[2]						
≤65 years	1,041	42.4	717	40.3	324	48.1
65-74 years	780	31.8	555	31.2	225	33.4
≥75 years	631	25.7	506	28.5	125	18.6
Missing	1		0		1	

[1] Percentages exclude missing data and may not add up to 100% due to rounding

[2] Age is calculated using date of birth and date of interview or survey participation; if date of participation is unavailable, the median date for survey participation was used (10/20/2018)
