# Supplementary material for: What Results Should Be Returned from Opportunistic Screening in Translational Research?
Source: J Pers Med. 2020 Mar 1;10(1):13. doi: 10.3390/jpm10010013 (PMC7151595; doi:10.3390/jpm10010013)
Supplement: Supplementary file 1 [file jpm-10-00013-s001.zip › supplementary files/Supplemental TABLE 2 final.pdf]

Supplemental Table 2. Participant demographics by survey responding status and result

	Non-responders Positive (N=187)		Non-responders Negative (N=1,591)		Responders Positive (N=36)		Responders Negative (N=639)	
	n	%[1]	n	%[1]	n	%[1]	n	%[1]
Sex								
Male	98	52.4	877	55.1	10	27.8	323	50.6
Female	89	47.6	714	44.9	26	72.2	315	49.4
Missing	0		0		0		1	
Race								
Non-Hispanic White	171	95.0	1,384	91.6	35	100.0	572	93.0
African American	8	4.4	108	7.2	0	0	33	5.4
Hispanic	1	0.6	19	1.3	0	0	10	1.6
Missing	7		80		1		24	
Age at participation[2]								
≤65 years	72	38.5	645	40.5	18	50.0	306	48.0
65-74 years	63	33.7	492	30.9	9	25.0	216	33.9
≥75 years	52	27.8	454	28.5	9	25.0	116	18.2
Missing	0		0		0		1	

[1] Percentages exclude missing data and may not add up to 100% due to rounding
[2] Age is calculated using date of birth and date of interview or survey participation; if date of participation is unavailable, the median date for survey participation was used (10/20/2018)
