# Supplementary material for: What Results Should Be Returned from Opportunistic Screening in Translational Research?
Source: J Pers Med. 2020 Mar 1;10(1):13. doi: 10.3390/jpm10010013 (PMC7151595; doi:10.3390/jpm10010013)
Supplement: Supplementary file 1 [file jpm-10-00013-s001.zip › supplementary files/Supplemental TABLE 3 final.pdf]

Supplemental Table 3. Participant demographics by survey responding status and result, excluding non-responders with no email address available (n=702)

	Responders Positive (N=36)		Non-responders Positive (N=133)		Responders Negative (N=639)		Non-responders Negative (N=943)	
	n	%[1]	n	%[1]	n	%[1]	n	%[1]
Sex								
Male	10	27.8	68	51.1	323	50.6	533	56.5
Female	26	72.2	65	48.9	315	49.4	410	43.5
Missing	0		0		1		0	
Race								
Non-Hispanic White	35	100.0	123	94.6	572	93.0	849	93.1
African American	0	0	6	4.6	33	5.4	51	5.6
Hispanic	0	0	1	0.8	10	1.6	12	1.3
Missing	1		3		24		31	
Age at participation[2]								
≤65 years	18	50.0	61	45.9	306	48.0	439	46.6
65-74 years	9	25.0	46	34.6	216	33.9	297	31.5
≥75 years	9	25.0	26	19.6	116	18.2	207	22.0
Missing	0		0		1		0	

[1] Percentages exclude missing data and may not add up to 100% due to rounding
[2] Age is calculated using date of birth and date of interview or survey participation; if date of participation is unavailable, the median date for survey participation was used (10/20/2018)
